# Nonsense suppression induced readthrough of a novel *PAX6* mutation in patient‐derived cells of congenital aniridia

**DOI:** 10.1002/mgg3.1198

**Published:** 2020-03-03

**Authors:** Xiaoliang Liu, Yuanyuan Zhang, Bijun Zhang, Haiming Gao, Chuang Qiu

**Affiliations:** ^1^ Department of Clinical Genetics Shengjing Hospital of China Medical University Shenyang China; ^2^ Department of Orthopaedics Shengjing Hospital of China Medical University Shenyang China

**Keywords:** congenital aniridia, nonsense suppression therapy, nonsense‐mediated mRNA decay, *PAX6*

## Abstract

**Background:**

Congenital aniridia is a severe ocular abnormality characterized by incomplete formation of the iris and many other ocular complications. Most cases are caused by the *paired box 6* (*PAX6*) gene mutations generating premature termination codons (PTCs).

**Methods:**

Ophthalmic examination was performed on a Chinese pedigree with congenital aniridia. The mutation was identified by targeted next‐generation sequencing. Nonsense suppression therapy was applied on patient‐derived lymphocytes. The *PAX6* expression was assayed by real‐time polymerase chain reaction and Western blot.

**Results:**

Complete aniridia was complicated with horizontal nystagmus, contract, foveal hypoplasia, and microphthalmia. A novel heterozygous c.702_703delinsAT (p.Tyr234*) mutation was found in exon 9 of *PAX6*, generating a PTC at the homeodomain. There were about 50% reductions of both full‐length PAX6 protein and *PAX6* mRNA in patient‐derived lymphocytes, indicating haploinsufficiency due to nonsense‐mediated mRNA decay. Ataluren (PTC124) and geneticin (G418) could induce about 30%–40% translational readthrough. Nonsense suppression therapy restored PAX6 protein to about 65%–70% of unaffected family controls.

**Conclusion:**

Our data expanded the genetic and phenotypic variations of congenital aniridia, and showed the therapeutic effect of nonsense suppression on this disease using patient‐derived cells.

## Graphical abstract

Nonsense suppression therapy partially restored PAX6 expression in patient‐derived cells carrying p.Tyr234* mutation. Both ataluren (PTC124) and geneticin (G418) restored the expression of PAX6 protein to about 65%‐70% of healthy level by Western blot.

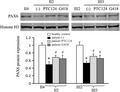

## 1 INTRODUCTION

Congenital aniridia (OMIM 106210) is a rare and severe ocular abnormality characterized by incomplete formation of the iris. It is also a panocular disorder by disrupted development in not only iris but also cornea, lens, optic nerve, anterior chamber, and retina (Schanilec & Biernacki, 2014). Familial cases show autosomal‐dominant inheritance pattern with high penetrance and variable expressivity (Chien et al., 2009). Some cases occur as part of Wilms tumor, aniridia, genitourinary abnormalities, and mental retardation syndrome (Fischbach, Trout, Lewis, Luis, & Sika, 2005).

The main genetic candidate for congenital aniridia is the *paired box 6* (*PAX6*) gene (OMIM 607108) on chromosome 11p13. It encodes a transcription factor that is involved in ocular morphogenesis (Ton et al., 1991). Approximately 72% of the *PAX6* mutations are frameshift, splicing site, or nonsense mutations that generate premature termination codons (PTCs; Tzoulaki, White, & Hanson, 2005). Premature termination codons usually elicit nonsense‐mediated mRNA decay (NMD) and cause haploinsufficiency for gene function (Kurosaki & Maquat, 2016). Nonsense suppression therapy with aminoglycosides and some other molecules could allow binding of near‐cognate tRNAs at PTCs and increase the translational readthrough for full‐length functional proteins (Keeling & Bedwell, 2011). The *Pax6*‐deficient (*Pax6* ^Sey+/−^) mouse model of aniridia showed elevated *Pax6* expression and reversed malformation defects in response to ataluren (PTC124) and geneticin (G418) treatment (Gregory‐Evans et al., 2014; Wang et al., 2017). There are currently no data about the therapeutic effect on aniridia patient‐derived cells.

In this study, we report a Chinese family with congenital aniridia carrying a novel in‐frame nonsense mutation of the *PAX6* gene. The *PAX6* expression was reduced in patient‐derived lymphocytes. The translational readthrough efficiency by nonsense suppression drugs PTC124 and G418 was assayed. Nonsense suppression therapy partially restored the expression of PAX6 in patient's lymphocytes.

## 2 MATERIALS AND METHODS

### 2.1 Ethical compliance

This study was approved by Ethics Committee of Shengjing Hospital of China Medical University.

### 2.2 Participants

A four‐generation Chinese family with congenital aniridia was included in this study. After obtaining written informed consents, peripheral blood samples were obtained from 12 family members for mutation analysis and four members for lymphocyte culturing.

### 2.3 Ophthalmic examination

Full ophthalmic examinations were performed on the proband (III3), including best‐corrected visual acuity, intraocular pressure, anterior segment evaluation using slit‐lamp microscopy, and fundus examination by optical coherence tomography. Patients II2 and IV2 also underwent most of the ophthalmic examinations except for anterior segment and fundus examination.

### 2.4 Sequencing analysis

Genomic DNA from peripheral blood leukocytes was isolated using the Blood Genomic DNA Miniprep Kit (Axygen). For the proband, a panel of 371 genes related to inheritable eye diseases was captured for targeted next‐generation sequencing. The genes were enriched using biotinylated capture probes (MyGenostics) as described previously (Jin et al., 2016), and were sequenced on the Illumina NextSeq 500 platform (San Diego, California, USA) with 50× coverage. Variant calling was performed with the Illumina NextSeq Reporter Software using NCBI37/hg19 assembly of the human genome as reference sequences. The mutation of *PAX6* was furthermore confirmed by Sanger sequencing in all the family members, with primers 5′‐TTGGTTGGAGGTAATGGGAGTGG‐3′ and 5′‐TGGCAGCAGAGCATTTAGCAGAC‐3′ (GenBank reference NG_008679.1). The polymerase chain reaction (PCR) products were sequenced using the Bigdye Terminator Cycle Sequencing Ready Reaction Kit and analyzed by ABI Prism 3730 Genetic Analyzer (Applied Biosystems).

### 2.5 Lymphocyte culture and drug administration

Lymphocytes from heparinized peripheral blood samples (II2, II4, III2, and III3) were collected by Ficoll‐Hypaque density gradient centrifugation, and cultured at 37°C and 5% CO_2_ using RPMI 1640 medium supplemented with 15% FBS, 1% glutamine, 5 mg/L phytohemagglutinin, 100 U/ml penicillin, and 100 μg/ml streptomycin. For drug treatment, cells were incubated in media containing PTC124 (Selleckchem) or G418 (Sigma) with indicated doses and exposure times. Cells with solvent (0.9% saline solution) treatment were used as controls.

### 2.6 Nuclear extraction and Western blot

Nuclear protein was extracted from lymphocytes using the Nuclear Protein Extraction Kit (Thermo Scientific). Protein concentrations were determined using Bradford method. Equal amounts were separated on an sodium dodecyl sulfate‐polyacrylamide gel electrophoresis gel, and transferred onto polyvinylidene fluoride membranes at 4°C. The membranes were subsequently blocked and incubated with primary antibodies against PAX6 (1:1,000; Abcam) and Histone H3 (1:3,000; Abcam) overnight at 4°C. Horseradish peroxidase‐conjugated IgG (1:5,000; Abcam) was used as a secondary antibody for incubation at room temperature for 2 hr. The final detection reaction was performed with the ECL detection kit (Thermo Scientific).

### 2.7 RNA isolation and real‐time quantitative PCR

Total RNA was extracted from lymphocytes using the RNeasy kit (Qiagen) according to the manufacturer's instructions. cDNA was synthesized using the Reverse Transcription Reagent Kit (Promega). Real‐time quantitative PCR was performed using SYBR Green method on the ABI 7500 System (Applied Biosystems), with primers specific to *PAX6* (GenBank reference NM_000280.4) 5′‐TTCAGATGAGGCTCAAATGC‐3′ (forward) and 5′‐CTGTATTCTTGCTTCAGGTAG‐3′ (reverse), and to *β‐actin* (GenBank reference NM_001101.5) 5′‐AACTGGGACGACATGGAGAAA‐3′ (forward) and 5′‐TAGCACAGCCTGGATAGCAAC‐3′ (reverse). Relative quantification of the *PAX6* mRNA was determined using comparative Ct method with the *β‐actin* transcript as an internal control.

### 2.8 Statistical analysis

Statistical analysis was run with the SPSS 16.0 software (SPSS). Data are presented as mean ± *SD* of three experiments. Statistical differences were determined by independent‐sample *t* test or one‐way ANOVA as appropriate. *p* < .05 was considered significant.

## 3 RESULTS

### 3.1 Pedigree and clinical characteristics

**Figure 1:**
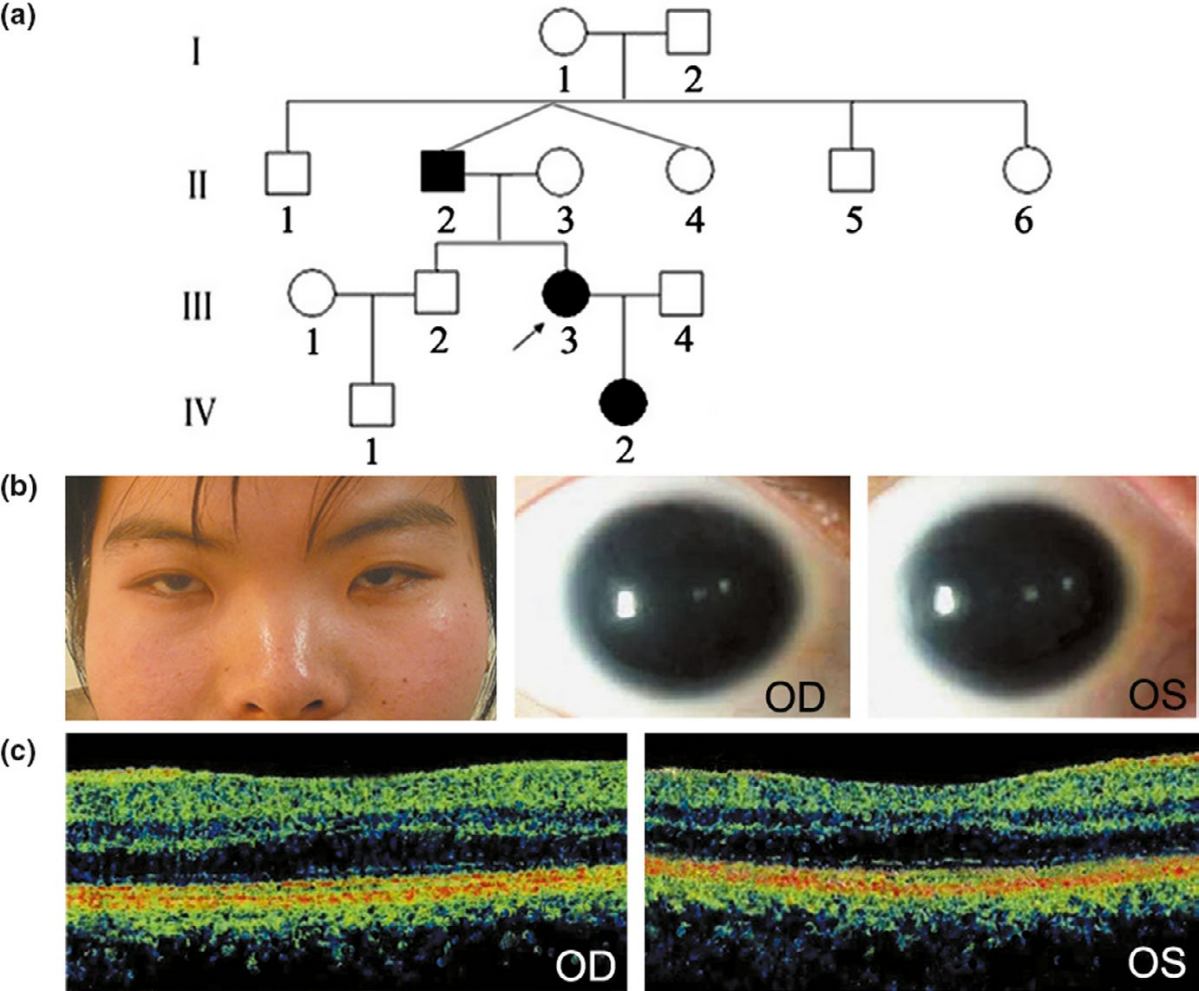
Pedigree and clinical manifestation. (a) Pedigree of the Chinese family with congenital aniridia. Affected individuals are depicted as black symbols; unaffected individuals are depicted as white symbols; the proband (III3) is indicated by an arrow. (b) Microphthalmia is shown in the regular face photograph of the proband. Bilateral complete aniridia is shown by anterior segment evaluation. (c) Foveal hypoplasia is shown by optical coherence tomography

The proband (III3) came to genetic counseling for the inherited aniridia in her family. The disease started from her father and was transmitted to her and her daughter in an autosomal‐dominant inheritance pattern. Her grandparents and her father's dizygotic twin sister were normal (Figure 1a).

The three affected patients in the family shared similar clinical symptoms including bilateral complete aniridia, cataract, horizontal oscillatory nystagmus, microphthalmia, foveal hypoplasia, photophobia, strabismus, and low visual acuity, with some degree of variations (Figure 1b,c; Table 1). Patients II2 and III3 had received cataract surgery. Relatively, patient II2 had slight corneal opacity; patient III3 had more serious cataract before surgery; whereas patient IV2 had more serious strabismus and photophobia. No other ocular or systemic abnormalities were found in the family.

**Table 1:** Clinical data of the affected family members with congenital aniridia

ID	Gender	Age	BCVA	IOP	Aniridia	Nystagmus	Cataract	Microphthalmia	Others
			OD/OS	OD/OS					
II2	M	58	0.2/0.1	20/21	+	+	±	+	Corneal opacity
III3	F	34	0.4/0.3	20/20	+	+	−	+	Foveal hypoplasia and photophobia
IV2	F	5	0.3/0.2	18/19	+	++	+	+	Strabismus and photophobia

Note

Age is shown as years; IOP is shown as mmHg; II2 and III3 had received cataract surgery.

Abbreviations: BCVA, best‐corrected visual acuity; F, female; IOP, intraocular pressure; M, male; OD, right eye; OS, left eye.

### 3.2 Mutation screening

**Figure 2:**
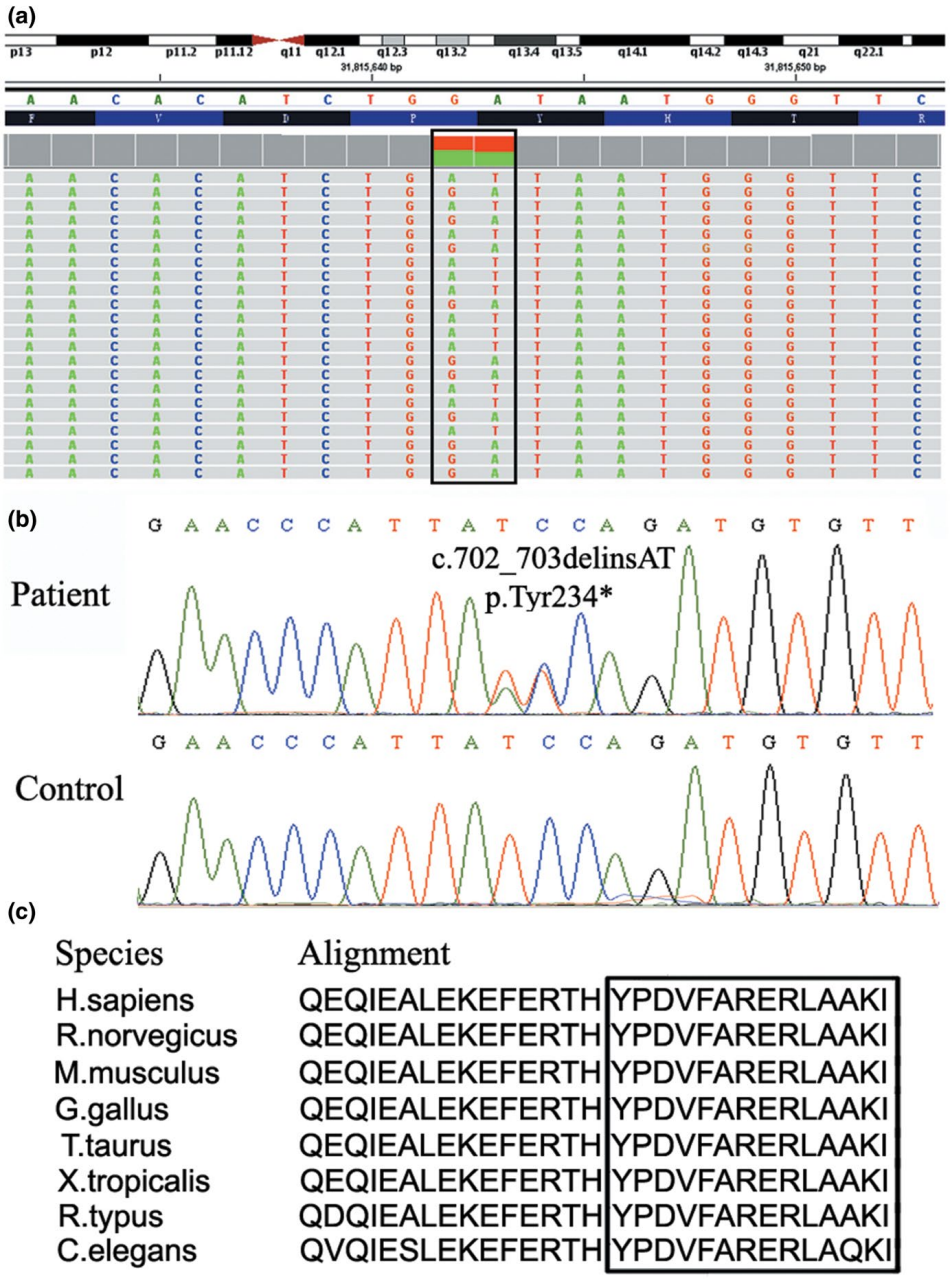
Sequencing analysis. (a) The mutation was screened by targeted next‐generation sequencing, showing a heterozygous c.702_703delinsAT variant of *PAX6* (boxed). (b) The mutation is positive in the patient but not in the control by Sanger sequencing. (c) The amino acid sequences were aligned among different species, showing the deletions (boxed) are conserved

Targeted next‐generation sequencing of genes related to inheritable eye diseases was performed on the proband III3. A heterozygous dinucleotide variant c.702_703delinsAT was found in exon 9 of *PAX6* (Figure 2a). The substitution generated a PTC at Tyr 234 (p.Tyr234*). We furthermore confirmed this variant by Sanger sequencing, showing positive in all the affected individuals but not in unaffected family members (Figure 2b). The mutation occurred at the homeodomain. The Tyr234 and its downstream sequences are highly conserved among different species (Figure 2c). This nonsense mutation has not been reported previously.

### 3.3 Haploinsufficiency of PAX6 in patient‐derived lymphocytes

**Figure 3:**
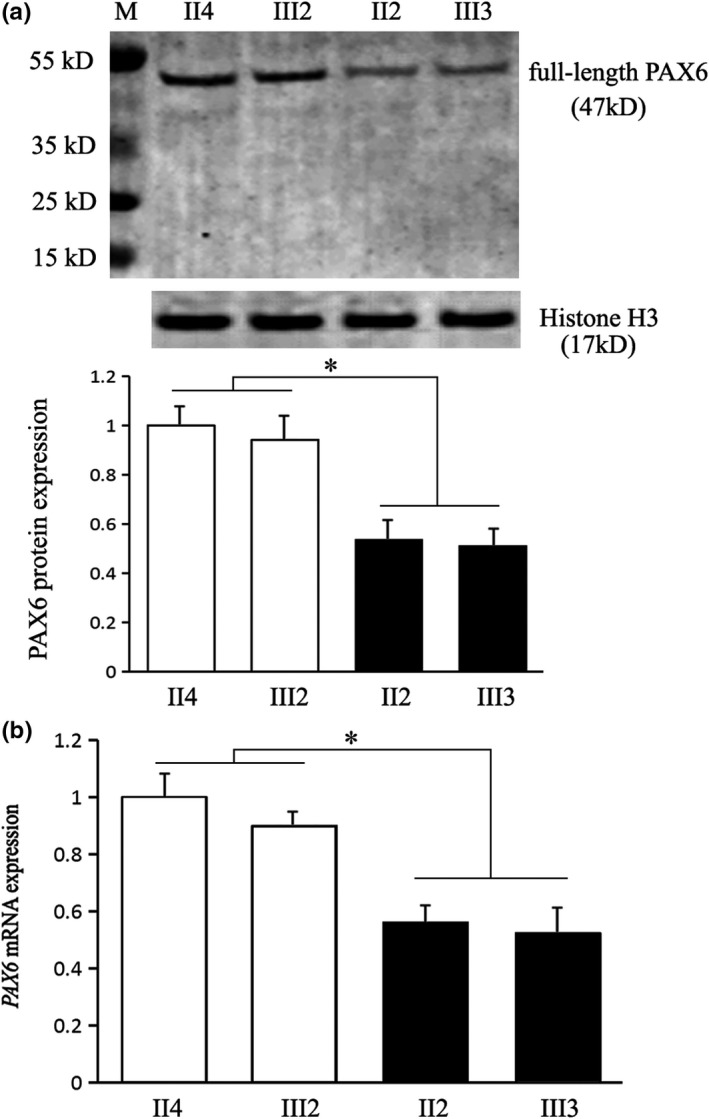
Haploinsufficiency of PAX6 in the patient‐derived lymphocytes. (a) By Western blot, there was about 50% reduction of full‐length PAX6 in the patients (II2 and III3) compared with the unaffected controls (II4 and III2; *p < .01). No truncated forms of PAX6 were observed. Histone H3 was used as an internal control. (b) The *PAX6* mRNA was analyzed by real‐time quantitative PCR, showing about 50% reductions in the patients (II2 and III3) than controls (II4 and III2; *p < .01). The *β‐actin* mRNA was used as an internal control. The level of individual II4 was taken as “1”

The effect of the novel nonsense mutation on PAX6 expression was studied in patient‐derived lymphocytes. The PAX6 protein in the nucleus was assayed by Western blot (Figure 3a). With Histone H3 as internal control, and with the relative level of a healthy family member as “1,” there were about 50% reductions of full‐length PAX6 protein in the patients (*p* < .01 vs. healthy control). We did not find truncated forms of PAX6 exclusively in the patients, indicating unstable truncation or NMD. The *PAX6* mRNA was evaluated by real‐time quantitative PCR (Figure 3b). Compared with the unaffected family controls, *PAX6* mRNA was also reduced to about half level in the patients (*p* < .01 vs. healthy control). Taken together, the p.Tyr234* nonsense mutation caused haploinsufficiency of PAX6 in the patients. A mechanism of NMD was involved.

### 3.4 Readthrough efficiency by nonsense suppression drugs

**Figure 4:**
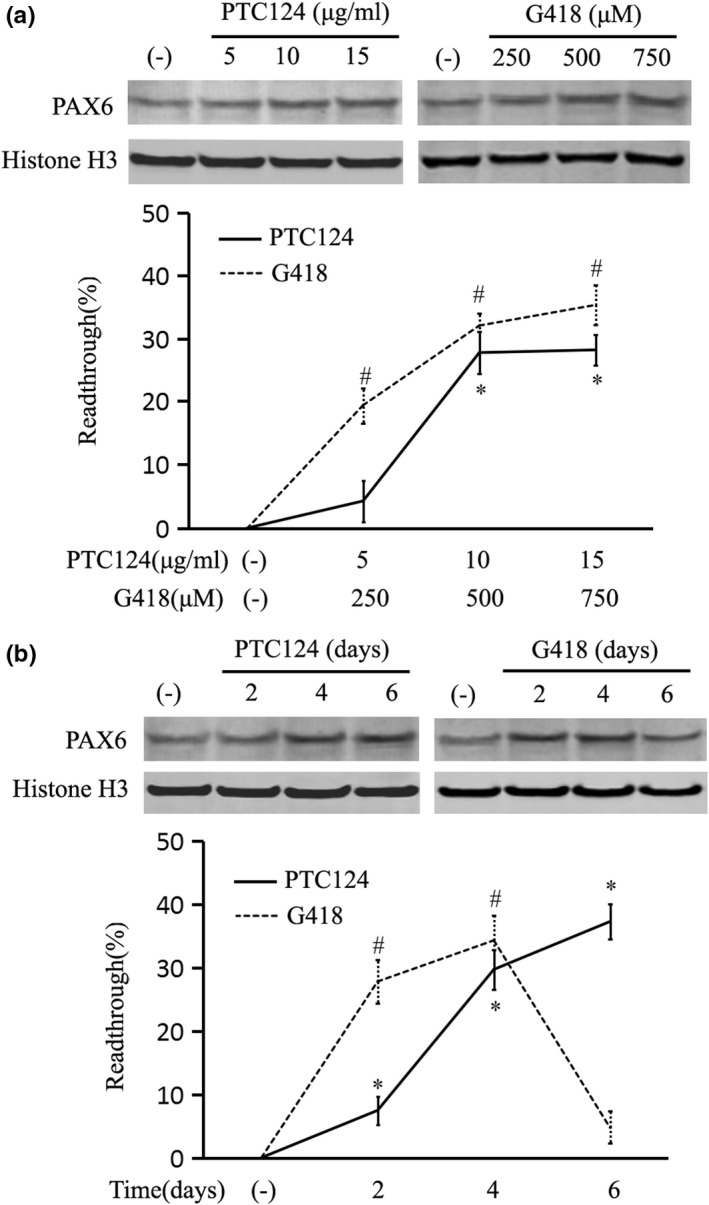
Readthrough efficiency of nonsense suppression drugs. The readthrough efficiency was calculated as a percentage of increased full‐length PAX6 over the solvent‐treated control (−) using lymphocytes of patient III3 by Western blot analysis. Histone H3 was used as an internal control. (a) The readthrough in response to varying doses of ataluren (PTC124) and geneticin (G418). Cells were harvested at four days of treatment for analysis. (b) The readthrough in response to varied treatment times, with optimized doses of 10 μg/ml PTC124 and 500 μM G418. *p < .01 versus (−) for PTC124; ^#^ *p* < .01 versus (−) for G418

We chose PTC124 and G418 to test the readthrough efficiency at the p.Tyr234* mutation in patient III3. Firstly, cells were treated with varying doses of drugs for four days. The readthrough efficiency was calculated as the increased percentage of relative full‐length PAX6 over solvent‐treated control by Western blot analysis. As shown in Figure 4a, the treatment of 5 μg/ml PTC124 only induced 4.27 ± 3.23% readthrough with no statistical significance. Strong inductions of readthrough were observed in cells treated with 10 and 15 μg/ml PTC124 (27.61 ± 3.27% and 28.03 ± 3.27%, *p* < .01 vs. solvent control). The readthrough remained almost unchanged with a higher dose of 15 μg/ml PTC124. For G418, the readthrough increased in response to 250, 500, and 750 μM treatments (19.53 ± 2.75%, 32.13 ± 2.04%, and 35.39 ± 2.92%, *p* < .01 vs. solvent control). However, cytotoxicity was observed at the higher dose of 750 μM G418.

Secondly, we selected 10 μg/ml PTC124 and 500 μM G418 to investigate the different treatment schedules (Figure 4b). The readthrough increased continuously with PTC124 treatment for 2, 4 and 6 days (7.48 ± 2.21%, 29.75 ± 3.14%, and 37.33 ± 2.79%, *p* < .01 vs. solvent control). As to G418, there was a rapid response with a satisfactory readthrough for 2 days treatment (27.78 ± 3.64%, *p* < .01 vs. solvent control). The readthrough furthermore increased to 34.27 ± 3.72% (*p* < .01 vs. solvent control) for 4 days treatment. However, due to significant cytotoxicity of 500 μM G418 for prolonged 6 days treatment, the readthrough drew back to nearly background level.

Taken together, there were about 30%–40% readthrough with limited cytotoxicity using 10 μg/ml PTC124 and 500 μM G418 for 4 days.

### 3.5 Recovered *PAX6* expression by nonsense suppression therapy

**Figure 5:**
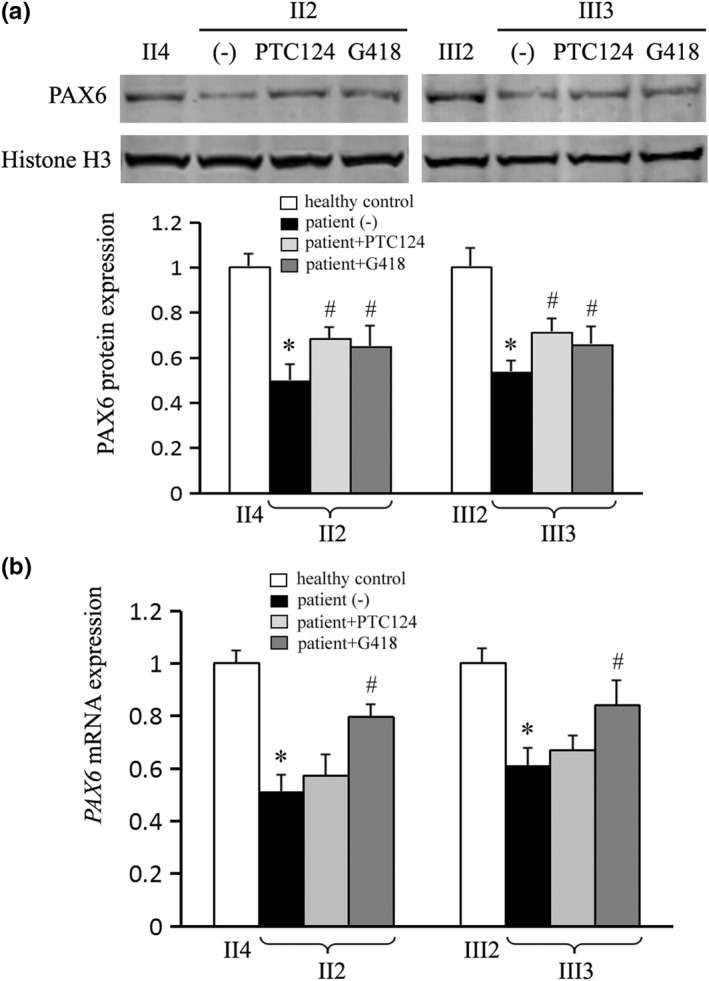
Recovered expression of *PAX6* by nonsense suppression therapy. Patients II2 and III3 were compared with the age‐matched II4 and III2 healthy controls, respectively (taken as “1”). (a) Both ataluren (PTC124) and geneticin (G418) restored the PAX6 protein to about 65%–70% of a healthy level by Western blot. Histone H3 was used as an internal control. (b) The *PAX6* mRNA expression was restored to about 80% of the healthy level in G418 but not PTC124 treatment by real‐time PCR analysis. The *β‐actin* mRNA was used as an internal control. *p < .01 versus healthy control; ^#^ *p* < .01 versus solvent control (−)

Based on the aforementioned readthrough efficiency, we assayed the recovery of PAX6 protein and mRNA in both patients II2 and III3 with 4 days treatments of 10 μg/ml PTC124 and 500 μM G418. Compared with the age‐matched controls II4 and III2 respectively, nuclear PAX6 protein was decreased to about 50% (*p* < .01 vs. healthy control) in the patients' solvent‐treated cells, and recovered to about 65%–70% (*p* < .01 vs. solvent control) of the healthy level in response to both drugs by Western blot (Figure 5a). Meanwhile, the *PAX6* mRNA was assayed by real‐time quantitative PCR analysis (Figure 5b), showing a more sensitive response to G418 treatment, with the recovery of up to about 80% of the healthy controls (*p* < .01 vs. solvent control) in both patients. PTC124 mildly restored the *PAX6* mRNA to levels with no statistical significance. Overall, we observed partial recovery of *PAX6* expression in patients' cells by nonsense suppression treatment, which might be of therapeutic significance.

## 4 DISCUSSION

Congenital aniridia is a rare and panocular disorder affecting most of the ocular structures. Besides partial or complete absence of iris, there are concurrences of several other eye abnormalities (Lim, Kim, & Kim, 2017). Foveal hypoplasia is almost always present, resulting in reduced visual acuity and pendular horizontal nystagmus. Cataract occurs in 70%–85% of aniridia patients. Glaucoma occurs in 30%–67% cases. Keratopathy occurs in approximately 20% cases. Optic nerve hypoplasia occurs in approximately 10% of patients. Microphthalmia occasionally happens in some cases. In the present study, we identified complete bilateral aniridia accompanied with cataract, horizontal oscillatory nystagmus, foveal hypoplasia, microphthalmia, photophobia, strabismus, and low visual acuity in the affected members, with some intra‐familial variabilities. Corneal opacity was only found in the elder patient. No glaucoma was found. We did not find Wilms tumor or other systemic symptoms in this family.

Congenital aniridia is associated with gene mutations of *PAX6* as well as *FOXC1*,* PITX2* and *PITX3*. Targeted next‐generation sequencing of 371 genes related to genetic eye diseases was performed on the proband to rapidly acquire the mutation. We found a novel heterozygous dinucleotide substitution c.702_703delinsAT in exon 9 of *PAX6*. Sanger sequencing on all the family members showed that the variation co‐segregated with the disease. PAX6 acts as a transcription regulator that is involved in ocular morphogenesis. The mutation generates a PTC at Tyr234 in HD, the DNA binding domain. Conservation analysis showed that the Tyr234 and afterward amino acids are conserved across evolutionary timescales.

The NMD is a surveillance mechanism that prevents cells from producing potentially deleterious truncated proteins by fast decay of mRNAs harboring a PTC (Kurosaki & Maquat, 2016). It is activated when the first stop codon of the open reading frame occurs more than 50–55 nucleotides upstream of an exon‐exon junction, or when the distance between the first stop codon and the poly(A) binding protein becomes abnormally long (Behm‐Ansmant, Gatfield, Rehwinkel, Hilgers, & Izaurralde, 2007; Silva, Ribeiro, Inácio, Liebhaber, & Romão, 2008). The PTC of *PAX6* in this study fits both in location. The full‐length PAX6 protein was reduced to about half‐level in the patients than controls. There were no obvious truncated forms of PAX6. Furthermore, there was also about half reduction of *PAX6* mRNA in the patients. Therefore, an involvement of NMD was confirmed, resulting in haploinsufficiency of PAX6. Our mRNA data are consistent with previously reported cases with *PAX6* c.112delC c.50dupA and c.765+1_765+2delGT mutations (Chen et al., 2013; Zhang et al., 2017).

Nonsense suppression therapy is under investigation to treat PTC‐derived diseases by suppressing translation termination to restore deficient protein function. In the presence of aminoglycosides or other nonsense suppressors, near‐cognate tRNAs competed with eukaryotic release factors for binding at PTCs, allowing the synthesis of full‐length protein by incorporating a substitutive amino acid (Keeling & Bedwell, 2011). The readthrough efficiency varies to the PTC types, with a predilection for UGA>UAG>UAA (Bidou, Allamand, Rousset, & Namy, 2012). In addition, the genetic context surrounding PTC also influences readthrough efficacy. The nucleotides U immediately upstream of the PTC (−1 position) and C immediately downstream of the PTC (+4 position) are easier for readthrough (Floquet, Hatin, Rousset, & Bidou, 2012; Manuvakhova, Keeling, & Bedwell, 2000). In this study, the mutation generates a UAA stop code with a context of −1U and +4U, which seemingly has less superiority in readthrough except for the −1U. However, we are encouraged by the studies on the *Pax6* ^Sey+/−^ aniridia mice with a naturally occurring *Pax6* p.Gly194* mutation, showing postnatal nonsense suppression caused great induction of Pax6 protein to about 80%–100% of wild‐type level and reversed the ocular malformations (Gregory‐Evans et al., 2014; Hill et al., 1991; Wang et al., 2017). Hereby, the readthrough efficiency was tested on patient‐derived cells using PCT124 and G418 in this study. The best readthrough was about 30%–40% with 10 μg/ml PTC124 and 500 μM G418 treatment for 4 days. However, there was a severe toxic effect of G418 at higher dose for longer time. As expected, the induced readthrough restored the PAX6 protein in the patients to about 65%–70% of healthy family controls. Over all, the sensitivity to readthrough agents in our model is quite high. A new work on six *CDKL5* nonsense mutations showed that aminoglycoside drugs efficiently suppressed all the tested nonsense mutations, regardless of surrounding −1 or +4 nucleotides (Fazzari, Frasca, Bifari, & Landsberger, 2019). Therefore, the rules of predicting readthrough efficacy are still not definitive. The mRNA secondary structures or interactions of multiple factors should also be considered. More data are still needed.

Readthrough drugs exert nonsense suppression *via* different mechanisms including but not limited to NMD suppression. G418 and PTC124 are two different kinds of readthrough reagents. Both drugs efficiently restored PAX6 protein to 65%–70% of the healthy level. However, the *PAX6* mRNA was accordingly restored by G418 but not PTC124. G418, as a member of aminoglycoside, is proposed to low down the efficiency of the cellular proof‐reading machinery and suppress NMD (Keeling & Bedwell, 2011). The obvious recovery of the *PAX6* mRNA supported a mechanism of NMD suppression by G418. PTC124, a small molecule drug with less toxicity than aminoglycoside, has been widely used and clinically evaluated in the therapy of PTC diseases (Peltz, Morsy, Welch, & Jacobson, 2013). The target of its readthrough activity is proposed to be the ribosome by promoting near‐cognate tRNAs insertion, but not mRNA stabilization (Roy et al., 2016). The less‐induced *PAX6* mRNA by PTC124 in our study also supported a minor role of NMD suppression.

Patient‐derived cells, with the “real” context of the patient's genetic background, should represent as a better model for in vitro studies of therapeutic potential than transfected cell lines with constructed vectors. Nonsense suppression has been studied on patient‐derived cells of diseases such as Rett syndromes (Popescu, Sidorova, Zhang, & Eubanks, 2010), cystic fibrosis (Pibiri et al., 2015), and retinitis pigmentosa (Grayson et al., 2002; Schwarz et al., 2014), with inconsistent readthrough outcomes. It should be taken into consideration the different genes and different cell lines. We are the first to study nonsense suppression on patient‐derived cells of congenial aniridia. One major limitation of our work is lacking experiments about the function of the readthrough PAX6 protein. The original amino acid Tyr234 is conserved across species, and readthrough therapy is at risk to change the nosense into missense mutations. Yet Roy's study has shown that the replacements for UAA by PTC124 and G418 are mostly likely Tyr (~52%) and Gln (~46%; Roy et al., 2016). Moreover, missense mutations of *PAX6* usually cause milder forms of disease (Lima Cunha, Arno, Corton, & Moosajee, 2019). Readthrough with the incorporation of substitutive amino acid still has great potential of clinical benefit. We will evaluate the effects of protein function in the future. Further studies on more types of *PAX6* nonsense mutation are still needed. Being a dose sensitive gene of *PAX6*, the partially restored expression might ameliorate the disease phenotype. The phase II clinical trial of Translarna™ for nonsense aniridia (NCT02647359) is ongoing and we are looking forward to a good result.

In conclusion, we identified a novel c.702_703delinsAT mutation of the *PAX6* gene in a Chinese family with congenital aniridia. The mutation generated a PTC that reduced *PAX6* expression via a mechanism of NMD. Nonsense suppression effectively induced readthrough and partially restored PAX6 expression in patient‐derived lymphocytes.

## CONFLICT OF INTEREST

The authors declare no conflict of interest.

## AUTHORS' CONTRIBUTION

X.L. designed the experiment, analyzed the data and drafted the manuscript. Y.Z. acquired the clinical data. B.Z. performed molecular experiments. H.G. cultured the cells. C.Q. oversaw the study and revised the manuscript.
